# *FOXP3* pathogenic variants cause male infertility through affecting the proliferation and apoptosis of human spermatogonial stem cells

**DOI:** 10.18632/aging.102589

**Published:** 2019-12-19

**Authors:** Qianqian Qiu, Xing Yu, Chencheng Yao, Yujun Hao, Liqing Fan, Chunyi Li, Peng Xu, Geng An, Zheng Li, Zuping He

**Affiliations:** 1 State Key Laboratory of Oncogenes and Related Genes, Shanghai Cancer Institute, Ren Ji Hospital, School of Medicine, Shanghai Jiao Tong University, Shanghai, China; 2 Hunan Normal University School of Medicine, Changsha, Hunan, China; 3 Department of Andrology, The Center for Men's Health, Urologic Medical Center, Shanghai Key Laboratory of Reproductive Medicine, Shanghai General Hospital, Shanghai Jiao Tong University, Shanghai, China; 4 Institute of Reproductive and Stem Cell Engineering, School of Basic Medical Science, Central South University, Changsha, China; 5 Reproductive and Genetic Hospital of CITIC-Xiangya, Changsha, China; 6 Fertility Center, Shenyang Dongfang Jinghua Hospital, Shenyang, Liaoning, China; 7 Department of Reproductive Medicine, Third Affiliated Hospital of Guangzhou Medical University, Guangzhou, China; 8 Renji-Med X Clinical Stem Cell Research Center, Ren Ji Hospital, School of Medicine, Shanghai Jiao Tong University, Shanghai, China; 9 Shanghai Key Laboratory of Reproductive Medicine, Shanghai, China

**Keywords:** FOXP3 pathogenic variants, male infertility, spermatogenesis failure, spermatogonial stem cells, proliferation and apoptosis

## Abstract

Genetic causes of male infertility that is associated with aging are largely unknown. This study was designed to identify novel pathogenic variants of *FOXP3* gene causing azoospermia. One homozygous (c.155 G > T) pathogenic variant of *FOXP3* was identified in nine non-obstructive azoospermia patients, and one heterozygous (c.691 C > A) of *FOXP3* was found in one non-obstructive azoospermia patient. Pedigrees studies indicated that the homozygous (c.155 G > T) *FOXP3* pathogenic variant was inherited, while heterozygous (c.691 C > A) *FOXP3* pathogenic variant was acquired. Human testis carrying pathogenic variant exhibited abnormal spermatogenesis. FOXP3 protein was expressed at a lower level or undetected in spermatocytes of mutant testis of non-obstructive azoospermia patients compared to obstructive azoospermia patients. FOXP3 stimulated the proliferation and inhibited the apoptosis of human spermatogonial stem cells, and we further analyzed the targets of FOXP3. We have identified two new pathogenic variants of *FOXP3* in non-obstructive azoospermia patients with high incidence, and FOXP3 silencing inhibits the proliferation and enhances the apoptosis of human spermatogonial stem cells. This study provides new insights into the etiology of azoospermia and offers novel pathogenic variants for gene targeting of male infertility.

## INTRODUCTION

Infertility, a serious health problem associated with aging, affects 10%–15% couples around the world, and male factors account for 50% of the cases [1]. It has been estimated that there are about 50 million of people with infertility in China. A healthy and mature sperm is essential for male fertility, and notably, gene pathogenic variants (PV) and/or defects result in spermatogenesis failure and male infertility. Spermatogenesis, namely the formation and maturation of spermatozoa, involves the self-renewal and differentiation of spermatogonial stem cells (SSCs), meiosis of spermatocytes and spermiogenesis of spermatids in the testis. Human spermatogenesis is an intricate process that is mediated by genetic factors of male germ cells, hormones, and paracrine factors [2]. Evolutionarily conserved orthologs among different species may be required during spermatogenesis with indicative function importance [3].

Genetic disorders account for around 30% of infertility by male factor, and they are thought to be responsible for the majority of idiopathic cases [4]. PV of crucial genes lead to abnormal spermatogenesis and eventual male infertility. It has been reported that PV stabilizing the Piwi proteins are directly responsible for male infertility by impairing histone to protamine exchange during spermiogenesis [5]. We have recently demonstrated that *P63* PV results in the abnormalities of mouse male germ cells and significant increase of apoptosis in these cells [6]. We have compared transcriptomic profiles of human normal male germ cells and identified several potential key genes with specific expression patterns involved in the regulation of human spermatogenesis [7]. Nevertheless, very little is known about the genetic defects or PV responsible for male infertility. Studies on the genetic elements that affect male infertility could add novel insights into understanding the etiology of male factor infertility and offer new targets for gene therapy of male infertility.

Transcription factor forkhead box protein P3 (FOXP3) belongs to the forkhead-winged-helix family of transcriptional regulators. FOXP3 is a well-recognized characteristic of regulatory T (T_reg_) cells that control immune tolerance and maintain immune homeostasis [8]. Although constitutively expressed in T_reg_ cells and first discovered as master gene for the immunosuppressive function of CD4^+^CD25^+^ T_reg_ cells, its expression has been found in other cell types [9]. In recent years, numerous breakthroughs in the transcriptional control and regulatory mechanisms and activities of FOXP3 have provided new therapeutic targets for autoimmune diseases and cancer [10].

*FOXP3* gene is located at the Xp11.23 [11], which is a critical locus, since males have only one chromosome X (genotype XY) and females (genotype XX) have only one active allele [12]. Consequently, *FOXP3* gene defect or mutation to the genome is adequate to cause cell transformation [13]. PV of the *FOXP3* gene result in the fatal lymphoproliferative disorder of the scurfy mice [14], and the ortholog of the *FOXP3* gene mutated in human causes a similar fatal disorder, namely the X-linked syndrome [15]. The association between *FOXP3* polymorphisms and the risk of idiopathic infertility and endometriosis has been analyzed in women [16]. Nevertheless, the understanding in the relationship of *FOXP3* and male infertility is quite limited. Recently, FOXP3 has been reported as a critical regulator in mouse fertility and the spermatogenesis of *FOXP3* mutant males is arrested prior to spermatid elongation [17, 18]. However, the actual function of FOXP3 and the influence of *FOXP3* PV in human infertility remain unknown.

In this study, we identified two types of PV of *FOXP3* (GenBank: NM_014009.3) in a large cohort of patients with non-obstructive azoospermia (NOA). By tracking these PV in unrelated consanguineous families, we have unveiled their inheritance patterns as likely the causes of human abnormal spermatogenesis and male infertility. Moreover, we have explored for the first time the functions of FOXP3 in regulation of human spermatogonial stem cells. This study thus could advance our understanding the etiology of male infertility and provide novel PV for gene targeting of male infertility.

## RESULTS

### *FOXP3* PV in patients with NOA

**Figure 1:**
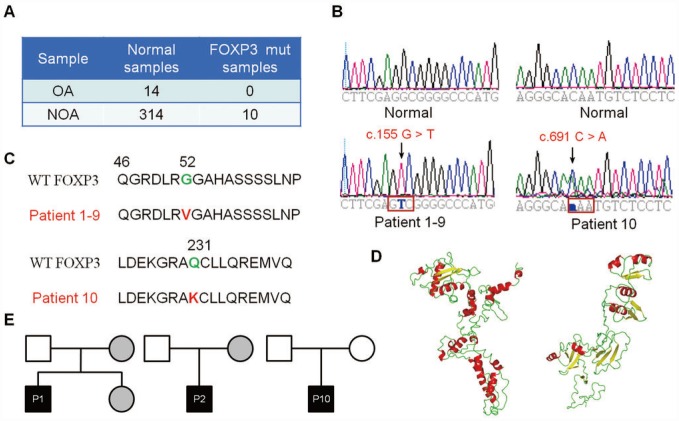
**Identification of *FOXP3* genomic PV from patients with NOA.** (**A**) A total of 314 patients with NOA and 14 OA patient controls were analyzed in this study. Ten PV of *FOXP3* were identified in 314 NOA patients (3.18% incidence, 10/314), whereas no PV of *FOXP3* was seen in the OA controls. (**B**) Chromatogram of the sequences in the mutant region of NOA patients and corresponding OA controls. (**C**) Comparison of the wild-type and mutant FOXP3 proteins. (**D**) FOXP3 structure model and stability prediction. (**E**) Pedigrees of Patients 1, 2 and 10 for their *FOXP3* PV. Circles, females; squares, males. Filled symbols, affected individuals; open symbols, unaffected individuals; gray symbols, carriers.

To explore the functional importance of FOXP3 in human male infertility, we screened for potential PV of *FOXP3* in 314 NOA patients and 14 OA controls (Figure 1A). Using exome sequencing, we identified the same variant in nine NOA patients (Patients 1–9) and another variant in one NOA patient (Patient 10) (Figure 1B and 1C); none of variant was detected in the OA controls (Figure 1B and 1C). The same variant was a homozygous missense PV in exon 1 (c.155 G > T (G52V)) (Figure 1B, left bottom panel, Figure 1C), while another variant was a heterozygous missense PV in exon 6 (c.691 C > A (Q231K)) (Figure 1B, right bottom panel, Figure 1C) in human *FOXP3* gene (GenBank: NM_014009.3). A full-length FOXP3 protein model (amino acid 1-431) was constructed (Figure 1D, left panel). FOXP3 may be processed by proteolytic cleavage upon cell activation, which leads to a truncated FOXP3 form (amino acid 52-417) (Figure 1D, right panel).

To track these PV in NOA patient pedigrees, we further sequenced the families of Patients 1, 2 and 10. Notably, the mothers of Patient 1 and Patient 2 and the fertile sister of Patient 1 shared the same heterozygous G52V PV, while their fathers carried the wild-type sequence (Figure 1E, left and middle panels), which reflects a recessive inheritance model. In contrast, both parents of Patient 10 presented wild-type *FOXP3* sequences (Figure 1E, right panel), indicating that Q231K arose de novo in this patient. These data suggest that both inheritable and de novo PV of *FOXP3* affect male fertility, and c.155 G > T PV of *FOXP3* is consistent with the X chromosome-linked inheritance patterns.

### Phenotypic and morphological characteristics of *FOXP3*-mut NOA patients

**Figure 2:**
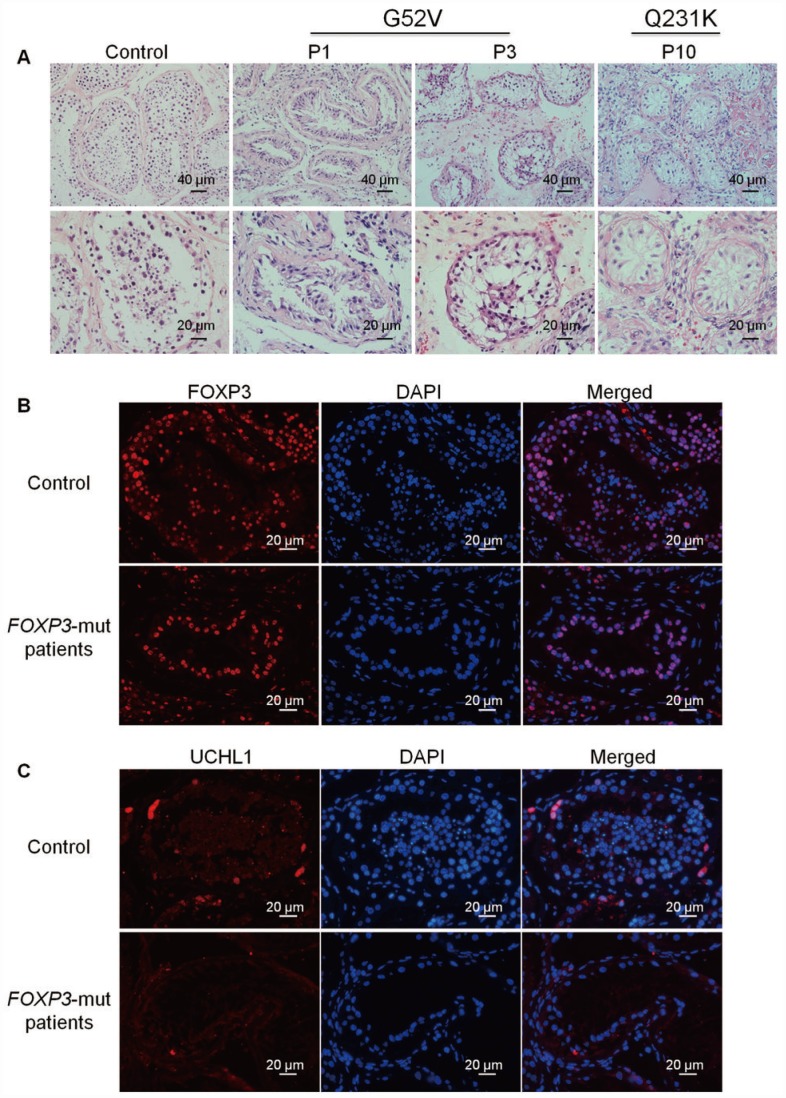
**Morphology and phenotype of *FOXP3*-mut NOA patients and OA controls.** (**A**) H&E staining revealed that seminiferous tubule diameter was reduced and spermatogenesis was arrested in *FOXP3*-mut NOA patients. Scale bars = 40 μm and 20 μm, respectively. (B-C) Immunohistochemical staining demonstrated the expression of FOXP3 protein (**B**) and UCHL1 protein (**C**) in *FOXP3*-mut NOA patients (low panels) and OA controls (upper panels). Experiments were repeated for at least three times. Scale bars = 20 μm.

We next evaluated the testicular morphology and determined whether intrinsic testicular defects occur in NOA patients when *FOXP3* was mutated. H&E staining of OA testis tissues demonstrated the normal seminiferous tubules containing different-stage male germ cells from SSCs to mature sperms (Figure 2A, left panels; Supplementary Figure 1A, left panels). Conversely, in the NOA testis with the PV of either G52V or Q231K, the diameters of seminiferous tubule were apparently reduced with arrested spermatogenesis (Figure 2A, middle & right panels; Supplementary Figure 1A, middle & right panels). Although spermatogonia and spermatocytes were present in *FOXP3*-mut testis of NOA patients, neither round haploid spermatids nor elongated spermatids were observed. In addition, we compared the expression level of FOXP3 protein in *FOXP3*-mut patients and OA controls, and we found that FOXP3 protein was expressed at a lower level or undetected in spermatocytes (Figure 2B; Supplementary Figure 1B). Intriguingly, level of UCHL1, a hallmark for human SSCs, was significantly decreased in *FOXP3*-mut testis compared to OA controls (Figure 2C; Supplementary Figure 1C). These results implicate a functional disorder of SSCs and spermatocytes caused by mutant FOXP3 protein.

**Figure 3:**
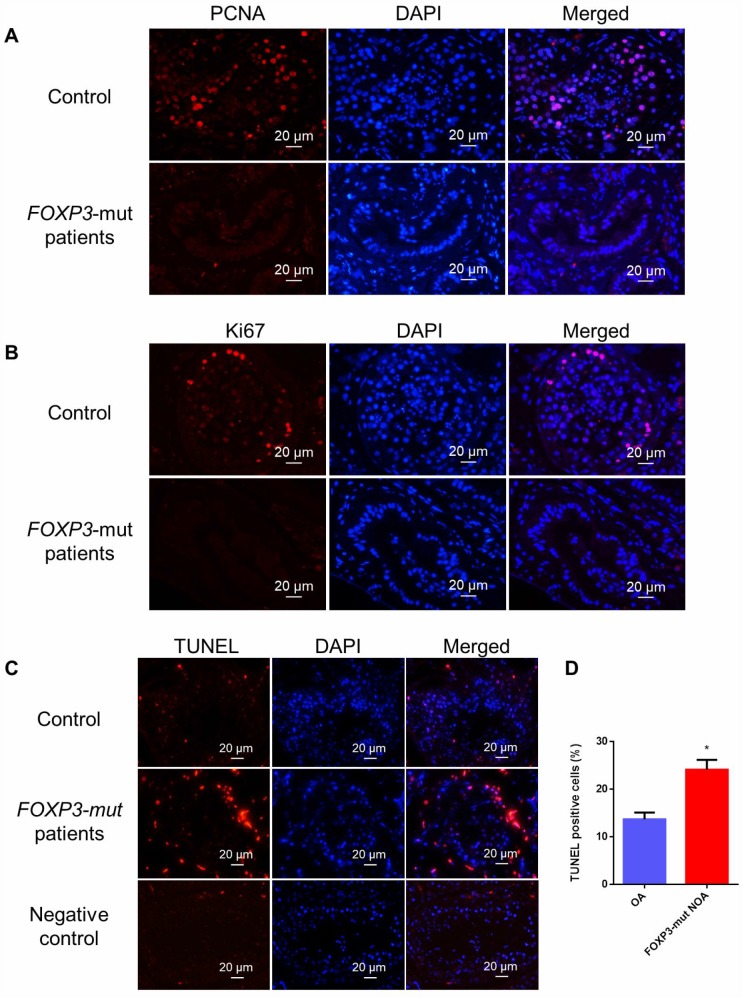
**Cell proliferation and apoptosis in the testis of *FOXP3*-mut NOA patients and OA controls.** (**A**, **B**) Immunohistochemical staining showed the levels of PCNA (**A**) and Ki67 (**B**), the hallmarks for cell proliferation, were decreased in *FOXP3*-mut NOA patients (lower panels) compared to the OA controls (upper panels). (**C**) TUNEL assay demonstrated the TUNEL-positive cells (red fluorescence) in *FOXP3*-mut NOA patients and OA controls. DAPI (blue fluorescence) was used to label cellular nuclei. Replacing the TdT enzyme with PBS was used as the negative control. Scale bars in A-C= 20 μm. (**D**) The percentages of apoptosis in male germ cells of *FOXP3*-mut NOA patients and OA controls were calculated using Student’s *t*-test. All values are means ± SD from three independent experiments. * indicated statistically significant differences (*p*<0.05).

In light of the decreased seminiferous tubule sizes and disrupted spermatogenesis in *FOXP3*-mut patients, we further assessed the overall proliferation and apoptosis levels of their testis tissues. Immunohistochemistry of two generally recognized markers of cellular proliferation, namely PCNA and Ki67, revealed that the proliferation of male germ cells was obviously reduced in *FOXP3*-mut testis compared to OA controls (Figure 3A, 3B; Supplementary Figure 2A, 2B), whereas the percentage of TUNEL-positive cells was significantly increased in *FOXP3*-mut testis compared with OA controls (Figure 3C–3D; Supplementary Figure 2C). Collectively, these data indicate that *FOXP3* PV results in abnormal testis morphology and spermatogenesis arrest in human by influencing the proliferation and apoptosis of male germ cells.

### FOXP3 expression in human testis tissues and SSCs

**Figure 4:**
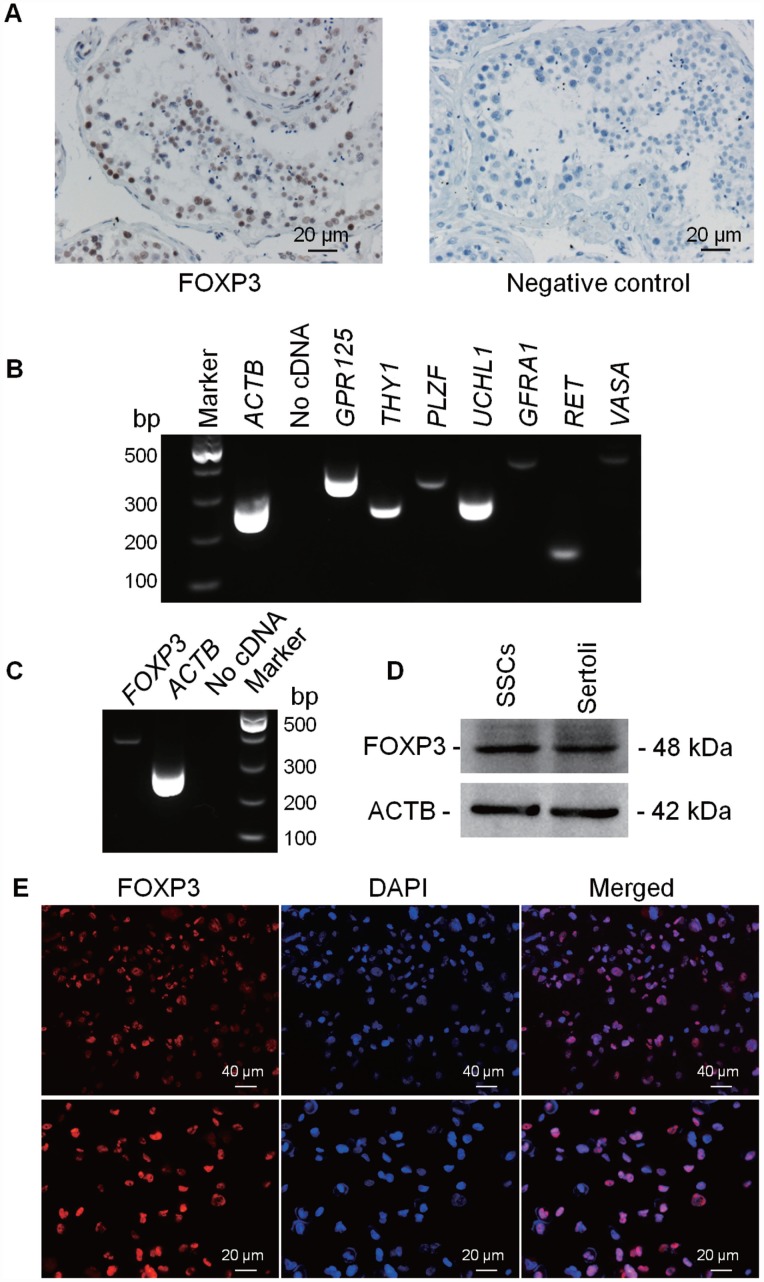
**Expression and location of FOXP3 proteins in human testes and human SSC line.** Immuno-histochemistry revealed cellular localization of FOXP3 in human testes (left panel). Replacement of anti-FOXP3 with PBS was used as a negative control (right panel). Scale bars = 20 μm. (**B**) RT-PCR showed the transcripts of *GPR125*, *THY1*, *PLZF*, *UCHL1*, *GFRA1*, *RET* and *VASA* in human SSC line. Samples without cDNA (No cDNA) but PCR with gene primers were used as negative controls. *ACTB* served as loading controls of total RNA. (**C**) RT-PCR showed the mRNA level of *FOXP3* in human SSC line. Samples without cDNA (No cDNA) but PCR with gene primers were used as negative controls, and *ACTB* served as a loading control of total RNA. (**D**) Western blots revealed the expression of FOXP3 protein in human SSC line. ACTB served as the control of the loading proteins. Human Sertoli cells were utilized as a positive control. (**E**) Immunocytochemistry revealed cellular localization of FOXP3 in human SSC line. Fluorescent signals of FOXP3 (red) and DAPI (blue) were imaged individually and merged under fluorescence microscope. Scale bars = 40 μm and 20 μm, respectively. All experiments were repeated for at least three times.

To determine cellular location of FOXP3 in human testis, immunohistochemistry was performed on human OA controls. FOXP3 protein was found to be extensively present in male germ cells, including SSCs, spermatocytes, and round spermatids of human testes, and the staining was especially strong in SSCs along the basement membrane (Figure 4A; Supplementary Figure 3A), which indicates functional importance of FOXP3 in mediating human SSC fate determination. Next, we verified the phenotype of our previously established human SSC line which expressed numerous genes for human germ cells, spermatogonia and SSCs, including *VASA, GPR125, GFRA1, UCHL1, THY1, RET* and *PLZF*, as shown by RT-PCR (Figure 4B; Supplementary Figure 3B). And the expression of SV40, GPR125, THY1, RET, DAZ2 and UCHL1 proteins have been previously demonstrated by Western blots and immunocytochemistry [19]. RT-PCR and Western blots showed that this SSC line expressed *FOXP3* both at mRNA (Figure 4C) and protein (Figure 4D) levels, and immunocytochemistry identified FOXP3 location in the nuclei of these cells (Figure 4E; Supplementary Figure 3C).

### Effect of FOXP3 on proliferation and apoptosis of human SSCs

**Figure 5:**
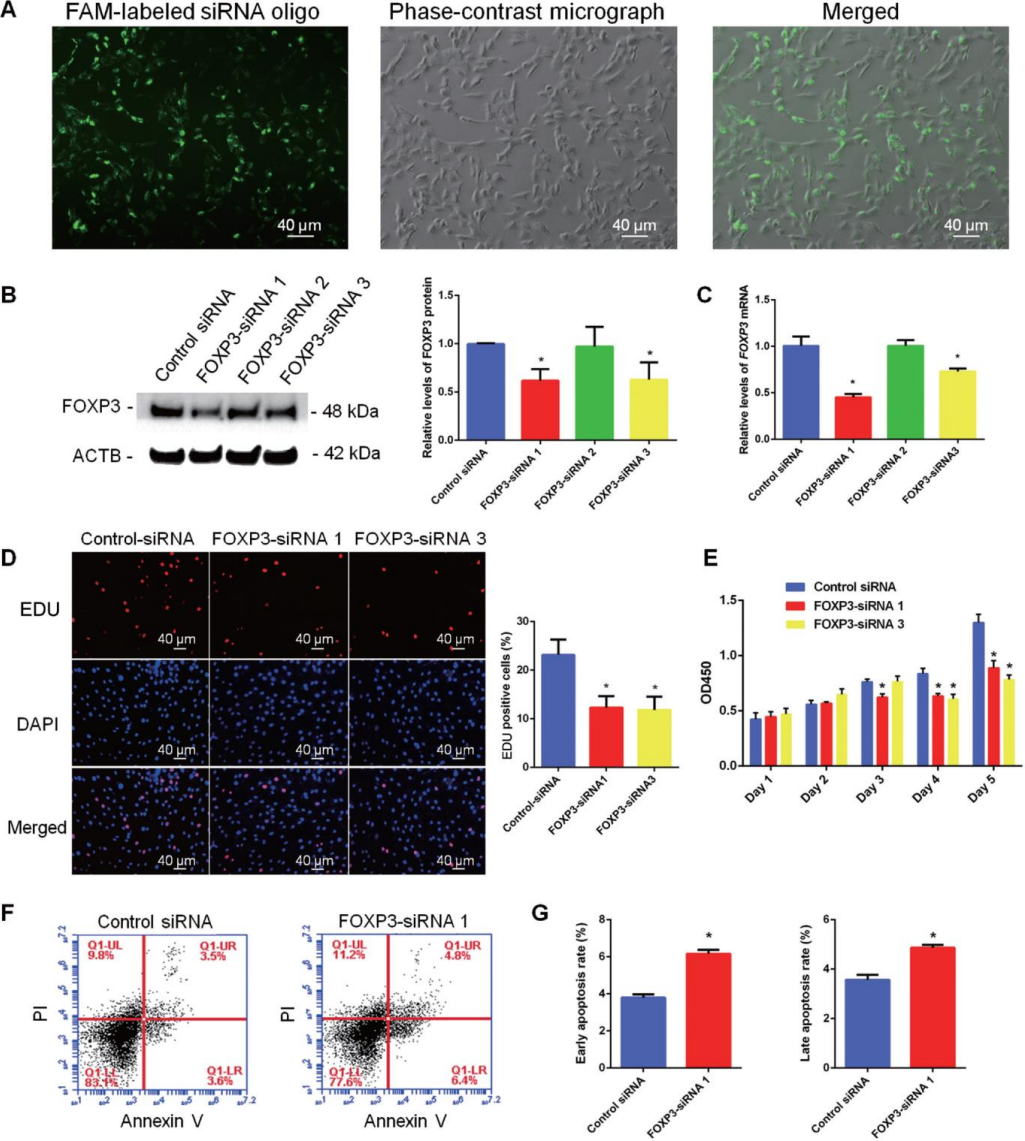
**Influence of FOXP3 Knockdown on the proliferation, DNA synthesis and apoptosis of human SSC line.** (**A**) Fluorescence microscope and phase-contrast microscope revealed transfection efficiency of FOXP3-siRNAs using the FAM-labeled miRNA oligonucleotides. Scale bars = 40 μm. (**B**) Western blots showed the protein changes of FOXP3 by FOXP3-siRNAs in human SSC line. (**C**) Real-time PCR displayed the mRNA changes of *FOXP3* by FOXP3-siRNAs in human SSC line. (**D**) EDU incorporation assay showed the percentages of EDU-positive cells affected by control siRNA, FOXP3-siRNA 1 and FOXP3-siRNA 3 in human SSC line. Scale bars = 40 μm. Values are means ± SD from three independent experiments. (**E**) CCK-8 assay demonstrated the proliferation of human SSC line after transfection with FOXP3-siRNA 1 and FOXP3-siRNA 3. (**F**–**G**) Annexin-V/PI staining and flow cytometry displayed the percentages of early apoptosis and late apoptosis of human SSC line transfected with FOXP3-siRNA 1 and control siRNA. All values are means ± SD from three independent experiments. * indicated statistical significance (*p*<0.05) between FOXP3-siRNAs and control siRNA treatments. All experiments were repeated for at least three times.

To further explore the function of FOXP3 in mediating the fate decision of human SSCs, we designed three pairs of small siRNAs targeting different regions of FOXP3 mRNA. FAM-labeled siRNA oligonucleotides indicated that the transfection efficiency of siRNA in human SSC line was around 80% (Figure 5A; Supplementary Figure 4A). Real-time PCR and Western blots revealed that both *FOXP3* mRNA and FOXP3 protein were significantly decreased in human SSC line by FOXP3-siRNA 1 and FOXP3-siRNA 3 (Figure 5B, 5C; Supplementary Figure 4B, 4C). Proliferation ability of human SSC line after knockdown of FOXP3 was determined by EDU and CCK-8 assays. The percentage of EDU-positive cells was diminished in human SSC line by FOXP3-siRNA 1 and FOXP3-siRNA 3 (Figure 5D; Supplementary Figure 4E). Consistently, CCK-8 assay showed that FOXP3-siRNA 1 and FOXP3-siRNA 3 inhibited the proliferation of human SSCs in a time-dependent manner (Figure 5E; Supplementary Figure 4D). Western blots displayed the decrease in PCNA level after knockdown of FOXP3 (Supplementary Figure 5). These data indicated that FOXP3 promotes proliferation and DNA synthesis of human SSCs. The apoptosis of SSCs after transfection of FOXP3-siRNA 1 and FOXP3-siRNA 3 was assessed by Annexin V-FITC/PI staining and flow cytometry demonstrating that FOXP3 silencing led to an increase of both early and late apoptosis of human SSC line, especially the early apoptosis (Figure 5F–5G; Supplementary Figure 4F). Taken together, these results suggest that FOXP3 promotes the proliferation and DNA synthesis whereas inhibits the apoptosis of human SSCs.

## DISCUSSION

Recent studies have advanced our understanding of autoimmunity-related infertility and therapeutic immunomodulators could favor pregnancy even in the cases of unexplained infertility [21]. As an indispensable molecule in immune tolerance, FOXP3 has been reported to play a critical role in mouse fertility since the PV of *Foxp3* leads to spermatogenesis arrest [17, 18]. Significantly, there are significant differences in cell types of male germ cells and stages between rodent and human spermatogenesis. Notably, hundreds of genes whose PV or defects have been identified to be essential for abnormal spermatogenesis in rodents are not applicable to humans. Herein, we initiated to screen the PV of *FOXP3* in clinical NOA patients and revealed the importance of FOXP3 in human male infertility. Two kinds of PV (c.155 G > T and c.691 C > A) in *FOXP3* gene were identified by us in NOA patients. The PV of *FOXP3* can either arise from hereditary factor or spontaneous change by environment, which may account for partially the etiology of idiopathic NOA. Identification of male infertility-associated PV in *FOXP3* and the investigation of its precise defects in molecular structures and functions resulting from specific PV could provide the definitive and causative link between FOXP3 dysfunction and male infertility in human.

FOXP3 protein is composed of three functionally important domains: a N-terminal domain, a zinc finger and leucine zipper-containing region, and a C-terminal forkhead (FKH) domain [10]. Multiple domains of FOXP3 are required for its functions as a transcriptional repressor. The FKH DNA binding domain is necessary for its nuclear localization and responsible for FOXP3-mediated transcriptional repressor functions by forming a ternary complex with the nuclear factor of activated T cells-1 (NFAT1) and a DNA oligonucleotide [22, 23]. The N-terminal domain can repress the transcription of targeting genes without binding DNA, and a functional domain within the N-terminal half is required for FOXP3-mediated repression of transcription. PV within the leucine zipper, which inhibits homodimerization of FOXP3, also reduces the repressor activity of FOXP3, indicating that homodimerization of FOXP3 is essential for FOXP3 function *in vivo* [23]. PV in any of the domains can lead to the scurfy or IPEX autoimmune phenotypes [10]. In this study, both of G52V and Q231K PV were predicted to influence the three-dimensional structure of FOXP3 protein. The *FOXP3* gene encodes a 47 kDa protein consisting of 431 amino acid [12], which may be processed by proteolytic cleavage to a shorter form (41 kDa) upon cell activation [24]. FOXP3 is cleaved at both the N- and C-terminal RXXR sites, and mutagenesis of the RXXR motif prevents cleavage [24]. Interestingly, the point of G52V is positioned right after the N-terminal cleavage site. G52V shows ddG of -0.63 in full length model and -1.9 in the truncated form, while Q231K demonstrates comparable ddG of -1.35 in the full length model and -1.1 in the truncated form. Both G52V and Q231K mutants seem to increase protein stability, which possibly hinders specific cell activation and biological process. Furthermore, those two PV also fall into potential recognition sites of TR KAP1, GABPA, SPI1 and ESR1.

Based upon these two models, we hypothesized that these PV may lead to dysfunction of male reproduction. We first compared the expression level of FOXP3 protein in male germ cells within human testis. Mutant testis of NOA patients expressed FOXP3 at a lower level compared to OA controls, indicating that these PV decrease general expression of FOXP3 in human male germ cells, especially human SSCs and spermatocytes. Moreover, we found that knockdown of FOXP3 inhibited the proliferation and induced the apoptosis of SSCs. Combining with the spermatogenesis disorders within the mutant testis tissue, we speculated that mutant FOXP3 breaks the balance between the proliferation and apoptosis of male germ cells, thus disturbing the spermatogenesis process.

We further unveiled the molecular mechanisms through which *FOXP3* PV impair spermatogonial stem cell function and human reproduction. Being a typical transcription factor, FOXP3 are highly possible to exert effect via its transcriptional regulatory activity or downstream targeting genes. As shown in the Supplementary Figure 6, we predict that FOXP3 interacts with a number of critical cytokines, such as IL-2, IL-6, IL-10 and TGFB1. Cytokines are important mediators of the immunity, and they can be involved in numerous processes in male genital tract, e.g. acting as immunomodulatory elements within the male gonad [25]. It has been reported that the levels of IL-2, IL-6, IL-10 and TGFB1 in seminal plasma or serum are significantly elevated in infertility patients compared to healthy controls [25–27]. Genetic deficiency of TGFB1 can cause male infertility and the infertility phenotype can’t be rescued by exogenous supplementation [28, 29]. On the other hand, IL-2 and TGFB are important for maintaining FOXP3 expression and activity [30, 31]. IL-6 can reduce FOXP3 transcription by triggering the methylation of the conserved non-coding sequence 2 (CNS2), whereas IL-2 has an opposite effect by recruiting demethylating TET enzymes [32]. The complex regulatory network connecting FOXP3 and cytokines may account for the dysfunction of mutant FOXP3 protein in male infertility. In addition, post-translational modification of FOXP3, including acetylation, phosphorylation and ubiquitylation, have recently been shown to be of great importance. A dynamic histone acetyltransferase-deacetylase complex is required for FOXP3-mediated transcriptional repression [33], and the acetylation of FOXP3 can modulate its intranuclear redistribution, posttranslational modifications, chromatin binding patterns and transcriptional suppressive activity [34]. Likewise, FOXP3 transcriptional activities are regulated by phosphorylation at Ser418 in the C-terminal DNA-binding domain [35], and PIM1 kinase-mediated phosphorylation of FOXP3 at serine 422 decreases its DNA binding activity [36]. FOXP3 protein is also subjected to ubiquitylation and deubiquitylation. It can be modified by the ubiquitin chains interlinked at lysine residue 48 (K48 polyubiquitylation) [37], and ubiquitylation negatively modulates cellular levels and stability of FOXP3 [38].

In summary, we have identified for the first time c.155 G > T (G52V) and c.691 C > A (Q231K) PV of *FOXP3* in NOA patients with 3.18% (10/314) of incidence, which may result in dysfunction of male reproduction and infertility. We have demonstrated that FOXP3 silencing inhibits the proliferation and DNA synthesis and enhances the apoptosis of human SSCs, and we further analyzed the targets of FOXP3. This study could offer new targets for the diagnosis and gene therapy of human male infertility.

## MATERIALS AND METHODS

### Human NOA and OA patients as well as peripheral blood and testis samples

In total, 314 patients with NOA were recruited from 4 hospitals, including Shanghai General Hospital affiliated to Shanghai Jiao Tong University, Reproductive and Genetic Hospital of CITIC-Xiangya, Oriental Jinghua Hospital of Shenyang, and The Third Affiliated Hospital of Guangzhou Medical University. Additionally, 14 obstructive azoospermia (OA) patients with normal spermatogenesis were recruited from Shanghai General Hospital affiliated to Shanghai Jiao Tong University. The written informed consent was obtained from all participants. Genomic DNA was extracted from the peripheral blood samples of NOA and OA patients. Testicular biopsies of patients identified with *FOXP3* mutant were further obtained to verify their PV in testicular genomic DNA.

### Exome sequencing

Exome sequencing was performed on genomic DNA of the OA and NOA patients with their families by Genewiz Co., Ltd. Primers designed to amplify each exon of the *FOXP3* gene were listed in Table 1. The TIANamp Genomic DNA Kit (TIANGEN, Beijing, China) was used for gDNA capture, and the PCR products were detected by agarose gel electrophoresis. Paired-end sequencing was conducted using ABI Prism^®^ 3730 instrument (Applied Biosystems, Foster City, CA, USA).

**Table 1:** Primer sequences used for whole exome sequencing of *FOXP3*

**Genomic DNA**	**Forward primers**	**Reverse primers**
Exon 1	TCTAGAGCTGGGGTGCAACT	CCCAGTGCCACAGTAAAGGT
Exon 2 and 3	CCATGAGCCTCAGTTTCCAT	CCAAGCCTCTGAGACCTGAC
Exon 4 and 5	TGGCCGTCTTTAAGCTTCTC	TATTGGGATGAAGCCTGAGC
Exon 6 and 7	GGGGCTCAGAGGAGAGAACT	CTCCCAAAGTGCTGGGATTA
Exon 8	CTTGCTTGAATCTGGGAGGT	CCGAAAGGAAGCTTTTGTGA
Exon 9 and 10	TTCAACCTCGGGGAGAACTA	ATGAGGGGTCACATTTGAGG
Exon 11	CCTGATTACCTGCCCCTACA	TGTGTTGAGTGAGGGACAGG

### Immunohistochemistry

Human testis paraffin sections were routinely deparaffinized and hydrated. After antigen retrieval and endogenous peroxidase blocking, the sections were blocked with 5% BSA (Sigma-Aldrich, St. Louis, MO, USA) for 1 hour at room temperature and incubated with primary antibodies, including anti-FOXP3 (Abcam, catalogue: ab22510), anti-UCHL1 (Abcam), anti-PCNA (Abcam), and anti-Ki67 (Abcam), at 4°C overnight. Antibodies binding were detected with HRP conjugated second antibodies, stained by DAB (Vector Lab, Burlingame, USA) and counterstained with hematoxylin, or detected by Alexa Fluor-conjugated second antibody (Invitrogen, USA) (1:200, room temperature, 1 h). DAPI was utilized for staining cell nuclei. The images were captured using the fluorescence microscope (Nikon, Tokyo, Japan).

### TUNEL

For testis paraffin sections, the general levels of apoptosis were evaluated via TUNEL Apoptosis Detection Kit (Alexa Fluor 647) (YEASEN, Shanghai, China) according to manufacturer’s instruction. After being deparaffinized and hydrated, human testis sections were permeabilized (Proteinase K, 20 min), equilibrated (1x Equilibration, room temperature, 30 min), incubated with TdT buffer (Alexa Fluor 647-dUTP Labeling Mix and TdT enzyme, 37°C, 1 h, in the dark), and counterstained with DAPI (room temperature, 5 min). Replacement of the TdT enzyme with PBS was used as the negative control, and the images was captured under fluorescence microscope.

### Cell culture and RNA interference (RNAi) of FOXP3

Human spermatogonial stem cell (SSC) line was established previously in our laboratory by transfecting primary human SSCs with a plasmid expressing the SV40 large T antigen [19], and this SSC line has been characterized with unlimited proliferation potentials and positive for a number of hallmarks for human spermatogonia and SSCs [19, 20]. Human SSC line was cultured (37°C, 5% CO_2_) in DMEM/F12 (Gibco Laboratories, Grand Island, NY) supplemented with 10% fetal bovine serum (FBS, Gibco, Thermo Fisher Scientific, Waltham, MA, USA) and 1% penicillin-streptomycin (Invitrogen, Carlsbad, CA, USA).

The FOXP3-siRNAs and control siRNA were synthesized by GenePharma (Suzhou, China), and the sequences of siRNAs were listed in Table 2. Cells were seeded at 0.8 x 10^5^/well density to 6-well plates, and transfection was performed using lipofectamine 3000 (Life technologies, Carlsbad, USA) according to manufacturer’s instruction. For each transfection, 4 μl of lipofectamine 3000 transfection reagent in 200 μl Opti-MEM (Invitrogen) were added to 200 μl of Opti-MEM predissolved with 120 pmol of siRNAs and incubated for 15 min at room temperature. The transfection efficiency was detected 6 h later using Nikon fluorescence microscope, and transfected cells were harvested at 24 h or 48 h for mRNA or protein analysis, respectively.

**Table 2:** The sequences for FOXP3 siRNAs

**siRNA names**	**Sense (5'-3')**	**Antisense (5'-3')**
FOXP3-siRNA 1	GGACACUCAAUGAGAUCUATT	UAGAUCUCAUUGAGUGUCCTT
FOXP3-siRNA 2	GUCUGCACAAGUGCUUUGUTT	ACAAAGCACUUGUGCAGACTT
FOXP3-siRNA 3	CUGCCUCAGUACACUCAAATT	UUUGAGUGUACUGAGGCAGTT
Negative control	UUCUCCGAACGUGUCACGUTT	ACGUGACACGUUCGGAGAATT

### RNA extraction, RT-PCR and quantitative real-time PCR

Total RNA was extracted from human SSC line using RNAiso Plus reagent (Takara, Kusatsu, Japan), and the concentrations and quality of isolated RNA were determined by Nanodrop (Thermo Scientific, Massachusetts, USA). Reverse transcription (RT) was conducted by the HiScript^@^II 1^st^ Strand cDNA Synthesis Kit (Vazyme, R212-02) according to the manufacturer’s instruction. The primers of genes for RT-PCR and real-time PCR were listed in Table 3. The PCR reactions were carried out using Premix Taq™ (TaKaRa Taq™ Version 2.0 plus dye), and the products were analyzed by agarose gels stained with GenGreen nucleic acid gel stain (Genview) and Image Analyzer ChemiDoc XRS^+^ (Bio-Rad).

**Table 3:** Primer sequences of genes used for RT-PCR and quantitative real-time PCR

**Genes**	**Primer sequences**	**Product size (bp)**	**Tm (°C)**
**Primer sequences of genes used for RT-PCR**			
*VASA*	F: GCAGAAGGAGGAGAAAGTAGTG R: CTCGTCCTGCAAGTATGATAGG	289	56
*GPR125*	F: GCGTCATTACGGTCTTTGGAA R: ACGGCAATTCAAGCGGAGG	199	60
*GFRA1*	F: CGGGTGGTCCCATTCATATC R: TGGCTGGCAGTTGGTAAA	411	60
*THY1*	F: ATCGCTCTCCTGCTAACAGTC R: CTCGTACTGGATGGGTGAACT	135	52
*PLZF*	F: CGGTTCCTGGATAGTTTGC R: GGGTGGTCGCCTGTATGT	317	54
*RET*	F: CTCGTTCATCGGGACTTG R: ACCCTGGCTCCTCTTCAC	126	56
*UCHL1*	F: AGCTGAAGGGACAAGAAGTTAG R: TTGTCATCTACCCGACATTGG	265	60
*FOXP3*	F: CCTCCACAACATGGACTACTT R: TCAGGGGCCAGGTGTAGGGT	325	54
*ACTB*	F: CATGTACGTTGCTATCCAGGC R:CTCCTTAATGTCACGCACGAT	250	58
**Primer sequences of genes used for quantitative real-time PCR**			
*FOXP3*	F: CTTCAAGTTCCACAACATGCGACC R: TAGATCTCATTGAGTGTCCGCTGC	102	59
*ACTB*	F: CACTCTTCCAGCCTTCCTTC R: GTACAGGTCTTTGCGGATGT	104	60

Real-time PCR reactions were conducted using PowerUp^TM^ SYBR^®^ Green Master Mix (Applied Biosystems, Woolston Warrington, UK) and StepOnePlus Real-Time PCR System (Applied Biosystems, Carlsbad, CA, USA). The relative expression levels of indicated genes were analyzed via the C_T_ (threshold cycle) value by normalizing with the housekeeping gene *ACTB* [ΔC_T_=C_T_(GENE)−C_T_(*ACTB*)], and the changes of gene expression after treatment compared to the control was calculated by formula 2^−ΔΔCT^ [ΔΔC_T_ =ΔC_T_(treated)−ΔC_T_(control)].

### Western blots

Human SSC line was collected 48 h after transfection with control siRNAs or FOXP3-siRNAs. After being lysed with RIPA buffer (BiotechWell, Shanghai, China) containing protease inhibitor cocktail (MedChemExpress, New Jersey, USA) for 30 min on ice, lysates were centrifugated (12,000 rpm, 4°C, 15 min), separated using 10% SDS-PAGE and transferred to PVDF membranes. The blots were probed with primary antibodies against FOXP3 (Abcam, catalogue: ab22510) or PCNA (Abcam) and incubated with HRP-labeled secondary antibodies (Abcam). HRP-labeled ACTB (Proteintech, catalogue: HRP-60008) was used as control. The blots were detected by Chemi-Doc XRS system (Bio-Rad Laboratories, Hercules, USA).

### Immunocytochemistry

Human SSC line was fixed with 4% paraformaldehyde (PFA, room temperature, 30 min), permeabilized with 0.5% Triton X-100 (Sigma), blocked with 5% BSA (room temperature, 1 h) and incubated with the primary antibodies against FOXP3 (Santa Cruz, sc-166212) at a dilution of 1:50 at 4°C overnight. Antibody binding was detected with Alexa Fluor-conjugated second antibody (Invitrogen, USA) (1:200, room temperature, 1 h) and the nuclei were stained with DAPI (room temperature, 5 min). After extensive washes with PBS, cells were observed under the fluorescence microscope.

### CCK-8 assay

Human SSC line was seeded at a density of 4,000 cells/well in 96-well microtiter plates and transfected with various kinds of siRNAs as mentioned above. The proliferation of the cells was detected by CCK-8 assay (Dojin Laboratories, Kumamoto, Japan) according to the manufacturer’s instruction. In brief, 10 μl of CCK-8 reagents and 100 μl of DMEM/F12 medium were mixed before being added to each well. After three hours’ incubation, the absorbance at the wavelength of 450 nm was measured by microplate reader (Thermo Scientific).

### EDU incorporation assay

After being transfected with FOXP3 siRNAs in 96-well plate for 24 h, human SSC line was incubated with 50 μM EDU (RiboBio, Guangzhou, China) for 12 h, fixed (4% PFA, room temperature, 30 min), neutralized (2 mg/ml glycine) and permeabilized (0.5% Triton X-100 room temperature, 10 min). After being washed with 0.5% tritonx-100 in PBS and incubated with 100 μl of 1x Apollo-Fluor (RiboBio, Guangzhou, China) (room temperature, 30 min, in the dark), cellular nuclei were stained with DAPI for 5 min. The percentages of EDU-positive cells were counted from more than 500 cells and three independent experiments were performed.

### Annexin-V/PI staining and flow cytometry

The apoptosis rates of transfected human SSC line was determined by the APC Annexin V Apoptosis Detection Kit with PI (Biolegend, London, UK) and detected by flow cytometry pursuant to the manufacturer’s instruction. Cells were seeded at 0.8x10^5^ cells/well in 6-well plates and harvested after transfection. Simultaneous staining with APC Annexin V and PI distinguished healthy cells (FITC^-^PI^-^), early apoptotic (FITC^+^PI^-^) and late apoptotic or necrotic cells (FITC^+^PI^+^).

### Statistical Analysis

All data were presented as mean ± SD from at least three independent experiments and analyzed by Student’s *t*-test using GraphPad Prism 6.01 Software. Normality and homogeneity of variances were checked prior to conduct Student’s *t*-test, and *P* < 0.05 was considered statistically significant.

## Supplementary Material

Supplementary Figures
