# Supplementary material for: FOXP3 pathogenic variants cause male infertility through affecting the proliferation and apoptosis of human spermatogonial stem cells
Source: Aging (Albany NY). 2019 Dec 19;11(24):12581–99. doi: 10.18632/aging.102589 (PMC6949051; doi:10.18632/aging.102589)
Supplement: Supplementary Figures [file aging-11-102589-s001..pdf]

SUPPLEMENTARY FIGURES

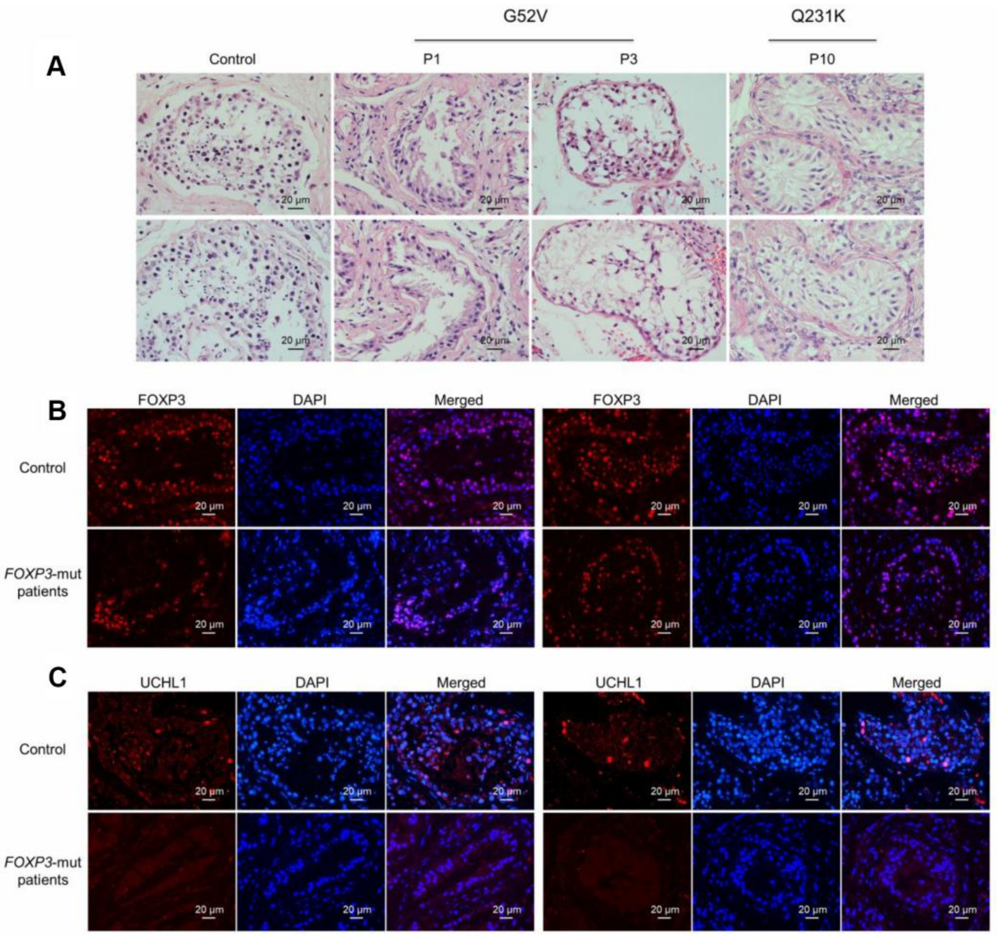

Supplementary Figure 1. Morphology and phenotype of *FOXP3*-mut NOA patients and OA controls. Representative data for the morphology of testis (**A**) and the expression of FOXP3 (**B**) and UCHL1 (**C**) in *FOXP3*-mut NOA patients and OA control.

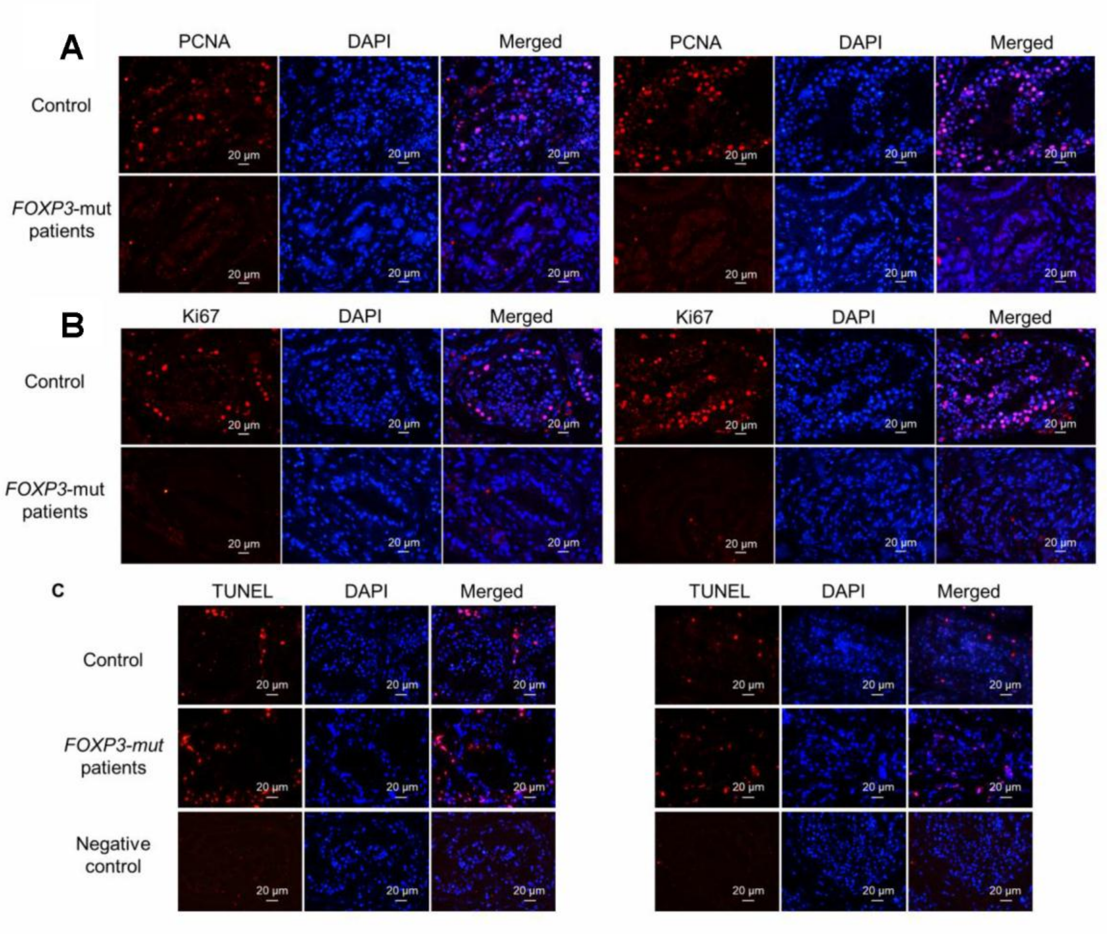

Supplementary Figure 2. Cell proliferation and apoptosis in the testis of *FOXP3*-mut NOA patients and OA controls. Representative results for the expression of PCNA (**A**), Ki67 (**B**) and TUNEL-positive cells (**C**) in *FOXP3*-mut NOA patients and OA control.

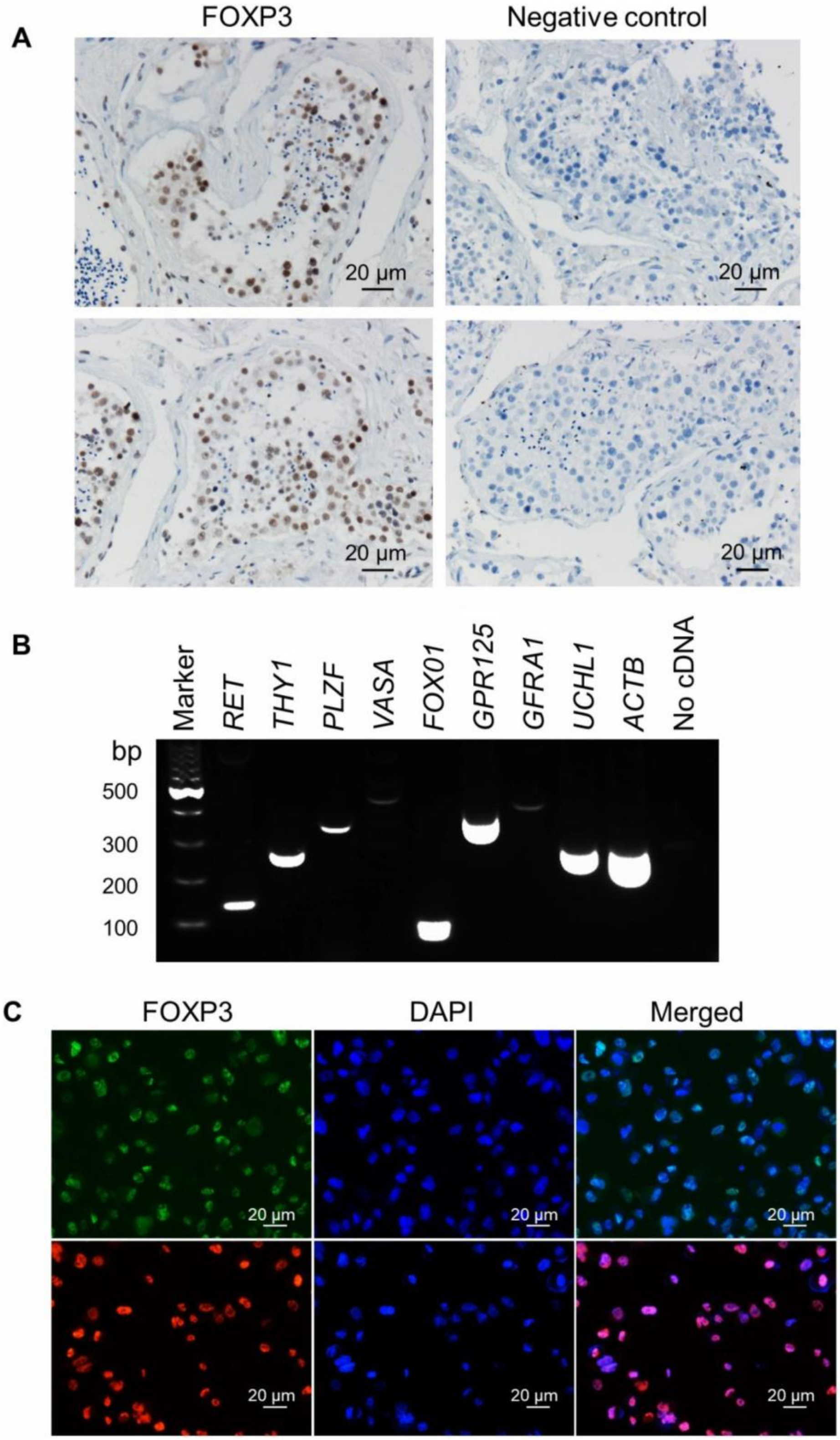

Supplementary Figure 3. Expression and location of FOXP3 proteins in human testes and human SSC line. Representative data for cellular localization of FOXP3 in human OA testes (**A**), the transcripts of *GPR125*, *THY1*, *PLZF*, *UCHL1*, *GFRA1*, *RET* and *VASA* in human SSC line (**B**), and FOXP3 expression in human SSC line (**C**).

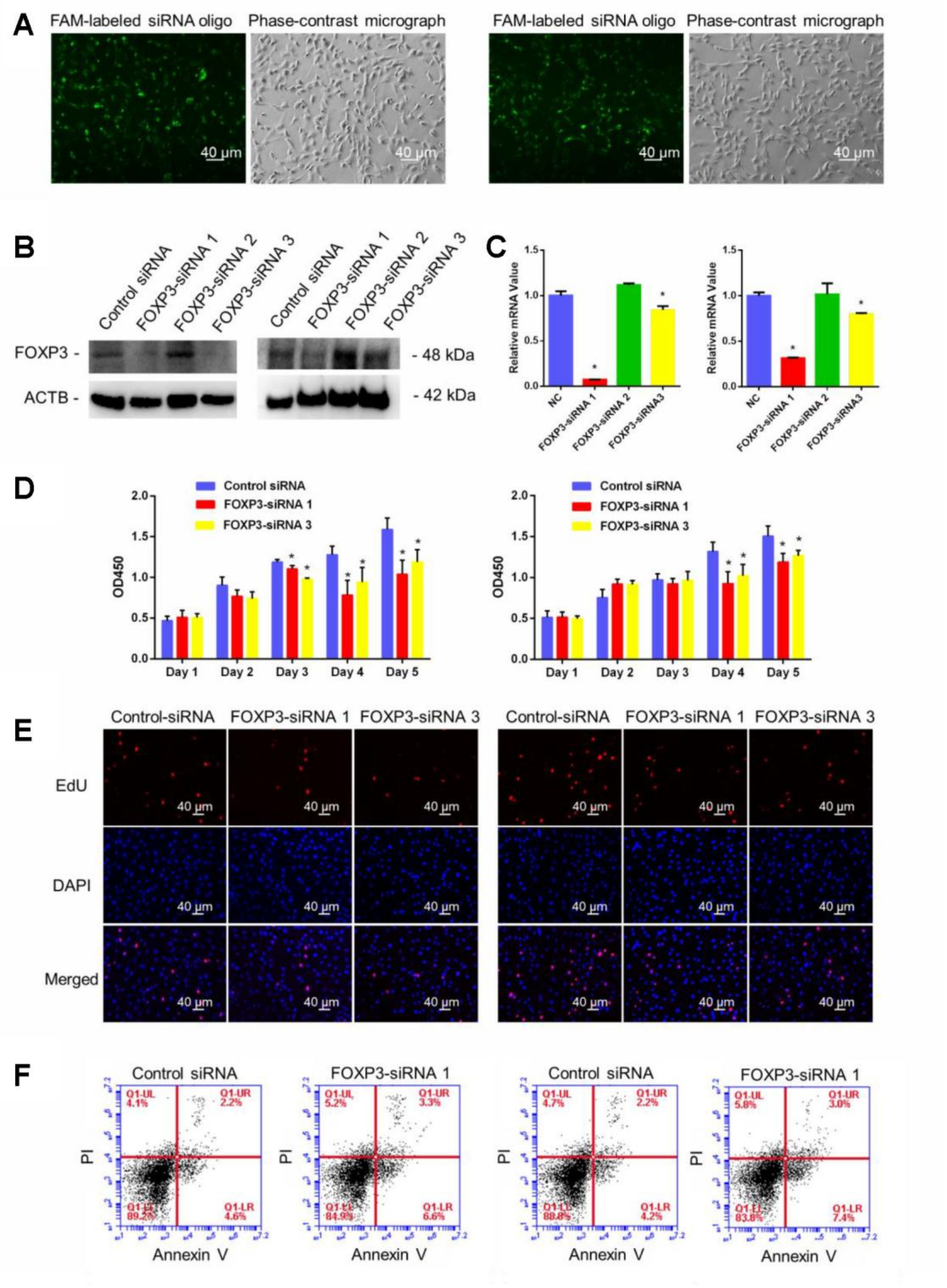

Supplementary Figure 4. Effect of FOXP3 silencing on the proliferation, DNA synthesis and apoptosis of human SSC line. Representative results for the transfection efficiency of FOXP3-siRNAs (**A**), the levels of FOXP3 protein (**B**) and *FOXP3* transcript (**C**) by FOXP3-siRNAs in human SSC line, as well as the percentages of the proliferation (**D**), EDU-positive cells (**E**) and apoptosis (**F**) by FOXP3-siRNAs in human SSC line.

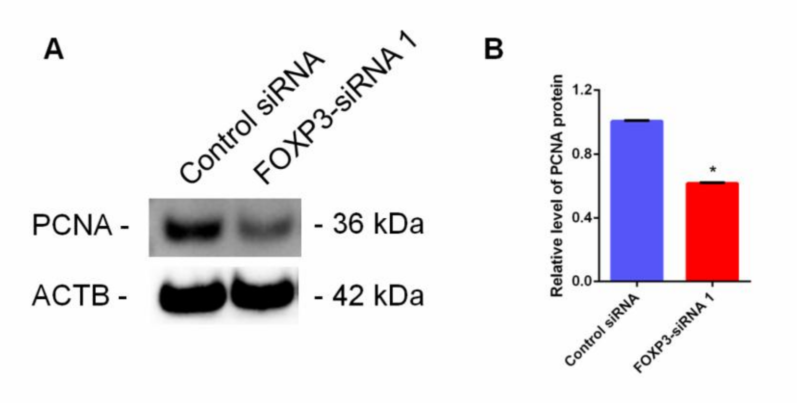

Supplementary Figure 5. Effect of FOXP3 on cell proliferation of human SSC line. (**A**) Western blots demonstrated PCNA expression in human SSCs at day 3 after transfection of FOXP3-siRNA 1. ACTB served as the loading control of protein. (**B**) The relative expression of PCNA in human SSCs at day 3 after transfection of FOXP3-siRNA 1 after normalization to the signals of their loading control. * indicated statistical significance ($p<0.05$) between FOXP3-siRNA 1 and control siRNA treatments.

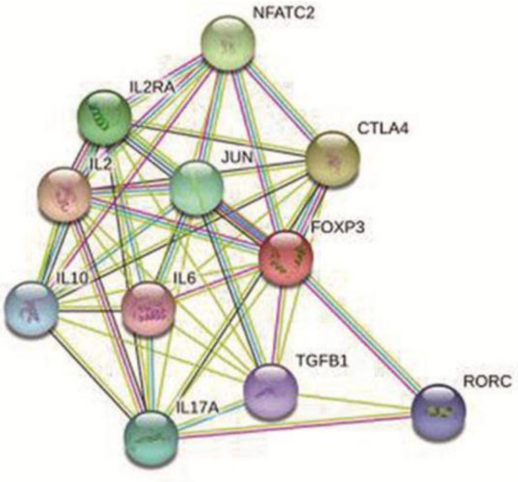

Supplementary Figure 6. Predicted partners and regulatory pattern of FOXP3. The main targets for FOXP3 were identified as ILs, TGFB1, CTLA4 and JUN.
